# Treadmill training for gait rehabilitation in elderly patients with mild-to-moderate Parkinson’s disease: a systematic review and meta-analysis

**DOI:** 10.3389/fneur.2025.1609912

**Published:** 2025-06-18

**Authors:** XiaoTing Yin, PeiQiang Peng, HongXia Zhang, JingYi Hu, YunHan Wei, PinMei Li

**Affiliations:** ^1^Department of Rehabilitation, Jilin University China-Japan Union Hospital, Changchun, China; ^2^Department of Rehabilitation, Zhejiang Provincial People's Hospital, Hangzhou, China

**Keywords:** Parkinson’s disease, treadmill training, lower extremity function, gait rehabilitation, elderly patients, meta-analysis

## Abstract

**Background:**

Parkinson’s disease (PD), the second most prevalent neurodegenerative disorder, leads to lower extremity dysfunction that critically contributes to falls and disability, yet effective rehabilitation remains limited.

**Objective:**

Systematic assessment of the effects of treadmill training on lower limb motor performance in patients with PD.

**Methods:**

As of March 1, 2024, a systematic search was conducted in PubMed, Web of Science, Embase, and the Cochrane Library to gather randomized controlled trials (RCTs) that report the effects of treadmill training on patients with PD. Data on the Unified Parkinson’s Disease Rating Scale Part III (UPDRS-III), the Timed Up and Go test (TUG), the Berg Balance Scale (BBS),6-Minute Walk Test (6MWT),10 Meter Walk Test (10MWT), and the Parkinson’s Disease Questionnaire-39 (PDQ-39) outcome metrics, as well as general characteristics of the studies, participant demographics, and details regarding the intervention and control groups, were extracted. The Cochrane Risk of Bias tool was employed to evaluate the quality of articles at risk, while the funnel plot and Egger’s test were utilized to assess publication bias.

**Results:**

16 RCTs comprising 582 participants were included. The meta-analysis indicated that treadmill training (TT) produced significantly better outcomes than conventional therapy (CT) in the post-intervention assessments of motor symptoms (UPDRS-III: SMD: -0.45; 95% CI: −0.73 to −0.17), and gait performance (6MWT: SMD 0.53; 95% CI: 0.08 to 0.97; 10MWT: 0.93; 95% CI: 0.54 to 1.32). Body Weight Supporting Treadmill (BBS) for Better Healing However, quality of life (PDQ-39: SMD: -0.35; 95% CI: −0.95 to 0.25), balance (BBS: SMD SMD: -0.35; 95% CI: −0.95 to 0.25; TUG: SMD: -0.35; 95% CI: −0.95 to 0.25), and treatment effects were comparable.

**Conclusion:**

TT (especially weight-supported) vs. conventional training demonstrates superior efficacy in enhancing lower limb mobility for Parkinson’s disease, improving muscular endurance and short-term gait speed, but requires enhanced dynamic balance integration.

**Systematic trial registration:**

https://www.crd.york.ac.uk/prospero/, identifier, CRD42021256958.

## 1 Introduction

PD is the second most prevalent neurodegenerative disorder globally, affecting approximately 3.6% of individuals over the age of 60 (1). The hallmark motor symptoms include resting tremor, rigidity, bradykinesia, and gait abnormalities, which progressively deteriorate as the disease advances, resulting in a substantial loss of functional independence (2).

Lower limb motor decline is a primary concern impacting patients’ quality of life (3). This condition is characterized by reduced muscle strength, restricted joint mobility, loss of gait symmetry, and impaired balance (4). These dysfunctions not only elevate the risk of falls—which can result in fractures, hospitalization, and even death, even in the early stages of the disease—but also further restrict physical activity, increase dependence, and contribute to psychological issues such as anxiety and depression (5).

Lower limb dysfunction is particularly pronounced in elderly patients with PD. This population frequently presents with comorbidities such as osteoporosis and arthritis, which can exacerbate the decline in lower limb motor function and compromise the safety of rehabilitation interventions (6). Additionally, the combined effects of age-related neuromuscular deterioration and the pathophysiology of PD further impair lower limb motor control (7). Consequently, developing targeted intervention strategies for elderly patients with PD is essential for enhancing their functional independence and overall quality of life.

The clinical management of PD is primarily dominated by pharmacological and neurosurgical treatments; however, physical therapy plays a crucial role in enhancing motor function (8, 9). In recent years, treadmill training has garnered attention for its dual mechanism: it not only improves lower limb muscle strength but also optimizes parameters such as stride length and step frequency through weight-bearing rhythmic exercise (10).

Additionally, it may promote neural plasticity in the motor cortex, thereby enhancing coordination (11). Although studies have shown improvements in gait and static balance, there are notable limitations in the existing evidence: (1) a lack of sufficient studies involving elderly patients with PD, which overlooks the impact of comorbidities on outcomes; (2) a fragmented assessment of lower limb capacity, which lacks an integrated analysis of muscle strength, gait, and functional activities.

Therefore, this study comprehensively assessed the effects of conventional/weight-loss treadmill training on lower limb motor abilities (e.g., lower limb muscle strength, endurance, balance and functional mobility) in elderly PD patients through systematic review and meta-analysis to provide an evidence-based basis for optimizing their rehabilitation strategies.

## 2 Methods

### 2.1 Protocol and registration

This systematic review was conducted in accordance with the Preferred Reporting Items for Systematic Reviews and Meta-Analyses (PRISMA) (12) guidelines. The protocol has been registered with the International Prospective Register for Systematic Reviews (PROSPERO; registration number CRD42021256958).

### 2.2 Literature search and study selection

A systematic search was conducted on March 1, 2025, across the following electronic databases: PubMed, Web of Science, Embase, and the Cochrane Library. The search strategy utilized the following keywords: (“Parkinson’s disease” OR “idiopathic Parkinson’s disease” OR “Lewy body Parkinson’s disease” OR “paralysis agitans” OR “primary parkinsonism”) AND (“treadmill training”). Only full-text articles published in English were included in the search.

### 2.3 Eligibility criteria

Two independent reviewers, Yin and Hu, screened the titles and abstracts of all retrieved records in a blinded manner. Any discrepancies were resolved by consulting a third reviewer, Li. Studies that met the screening criteria were subsequently evaluated in full text. All studies were included based on the following criteria:

Inclusion criteria: (1) Study design: randomized controlled trials (RCTs); (2) Population: Patients with Parkinson’s disease (Hoehn-Yahr stages I-III) aged ≥55 years; (3) Population: Disease duration ≥1 year, MMSE ≥24, and medically stable; (4) Intervention: conventional or body-weight-supported treadmill; (5) Comparison: Usual care, gait training, or standard comprehensive training; (6) Outcome: Includes UPDRS-III, BBS and TUG test, gait performance as assessed by 6MWT, 10MWT and quality of life as evaluated by PDQ-39.

Exclusion criteria: (1) Intervention: Combined with additional interventions; (2) Intervention: Short intervention duration (single or few sessions); (3) Comparison: Baseline data imbalance (Supplementary Table 1).

### 2.4 Data extraction

Data extraction was conducted independently by two reviewers, Yin and Hu. Any disagreements were resolved through discussion with a third reviewer, Li.

The extracted data included the following: (1) the general characteristics of each study (Authorship, Study design, Country, and Date of publication); (2) Characteristics of Participants (Age, Groups and Sample Size, Number of Men and Women, Parkinson’s Stage); (3) Characteristics of the intervention and control groups (treatment regimen, type of treadmill equipment): (4) Post-intervention quantitative data for the UPDRS-III, 6MWT, 10MWT, TUG, BBS, and PDQ-39, including mean and standard deviation.

Only data collected immediately after the intervention were included; no follow-up data were considered.

### 2.5 Risk of bias

Two reviewers (Yin and Hu) independently assessed potential bias using an improved Cochrane Risk of Bias Tool across five domains: randomization, intended interventions, missing outcome data, measurement of the outcome, and selection of the reported result. Each domain was categorized into one of three levels: high risk of bias, cause for concern, or low risk of bias. The reviewers consulted a third author, Li, to resolve any discrepancies.

### 2.6 Level of evidence

#### 2.6.1 Grading of recommendations, assessment, development, and evaluation (GRADE) rating of evidence quality

The GRADE system categorizes the quality of evidence based on five factors: bias. Risk, inconsistency, imprecision, indirectness, and publication bias are significant factors that can affect the quality of evidence. Quality was classified as high, moderate, low, or very low. The recommendations were divided into strong and weak levels.

#### 2.6.2 Oxford centre for evidence-based medicine: levels of evidence

Evidence levels were determined based on the latest guidelines from the Oxford Centre for Evidence-Based Medicine’s Evidence-Based Medicine Charts.

### 2.7 Statistical analysis

Statistical analyses were conducted using STATA version 15.1 (STATA Corp., College Station, TX, USA). The standardized mean difference (SMD) was employed to combine data, and Hedge’s g and 95% confidence intervals (CIs) served as effect size measures for continuous data. Heterogeneity among studies was evaluated using the χ^2^ test. An I^2^ ≤ 50% and *p* > 0.1 indicated no significant heterogeneity, while an I^2^ > 50% and *p* < 0.1 indicated significant heterogeneity. Potential sources of heterogeneity were investigated through subgroup analysis, and publication bias was assessed using funnel plots and Egger’s test.

## 3 Results

### 3.1 Search results

**Figure 1:**
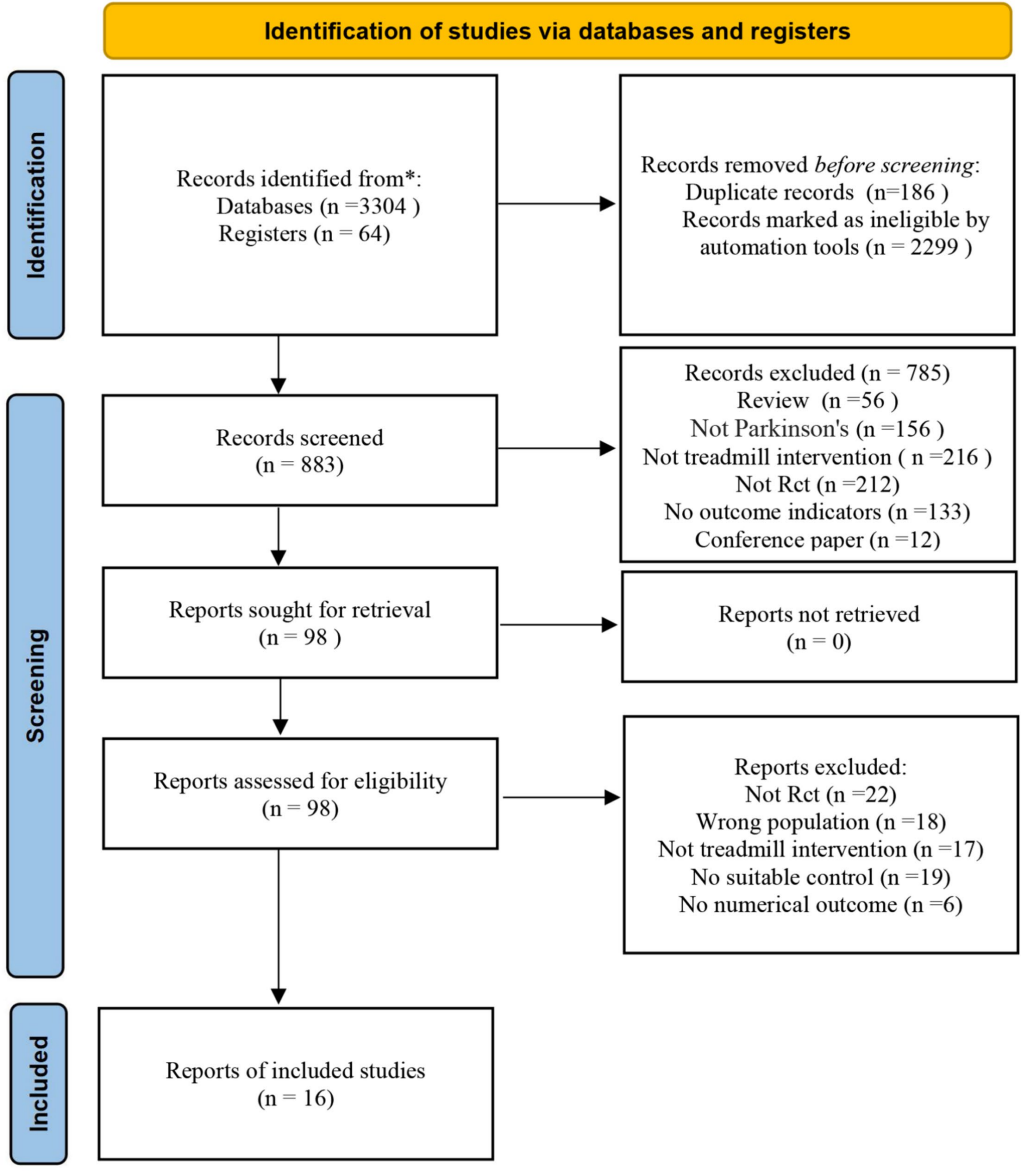
PRISMA flow diagram corresponding to the study selection process.

The initial search yielded 3,368 potential articles. Of these, 186 duplicates were excluded from the analysis. Additionally, 2,299 records were marked as ineligible by automation tools. After screening the titles and abstracts,98 studies were identified for further evaluation. Of these, 82 articles did not meet the inclusion criteria and were excluded from the study, leaving 16 studies for the meta-analysis (13–26) (Figure 1).

### 3.2 Study characteristics

A total of 16 RCTs (2000–2022) involving 582 participants were included. The treadmill group comprised 274 participants, while the control group included 308 participants. The study populations were from Japan, Turkey, the United States, Australia, Italy, India, China, Brazil, Iran, and Germany. The majority of participants were elderly aged 60 and above, with Hoehn-Yahr stages ranging from I to III. The treadmill group underwent either treadmill training (TT) or body-weight-supported treadmill training (BWSTT), while the control group primarily engaged in routine activities, gait training, or comprehensive training. The intervention duration ranged from 2 to 10 weeks, with a frequency of 2–4 sessions per week, each lasting 25–45 min (As shown in Tables 1, 2).

**Table 1:** General characteristics of the included studies.

Study	Type	Country	Age (y)	Gender (M? F)	H&Y staging	Treadmill brand
Miyai et al. (18)	RCT	Japan	67.6 ± 1.6	NA	II-III	NA
Miyai et al. (17)	RCT	Japan	69.6 ± 1.7	10/10	II-III	NA
Cakit et al. (39)	RCT	Turkey	71.8 ± 6.4	NA	II-III	NA
Fisher et al. (24)	RCT	United States	62.87 ± 11.87	11/9	I-II	NA
Canning et al. (40)	RCT	Australia	61.8 ± 8.23	11/9	I-II	NA
Picelli et al. (16)	RCT	Italy	68.28 ± 8.32	14/26	III	Jog Now 500MD
Harro et al. (19)	RCT	United States	66.10 ± 10.31	13/7	I-II	NA
Ganesan et al. (22)	RCT	India	58.15 ± 8.7	30/10	1-III	Gait Trainer
Ganesan et al. (23)	RCT	India	58.13 ± 9.28	46/14	1-III	Gait Trainer
Ganesan et al. (21)	RCT	India	58.15 ± 8.7	NA	1-III	Biodex Medical Systems
Picelli et al. (15)	RCT	Italy	67.24 ± 8.2	9/8	1-III	Jog Now 500MD
Cheng et al. (26)	RCT	China	84.8 ± 9.21	17/7	1-III	Rmax Science & Technology Co. Ltd.
Schenkman et al. (14)	RCT	USA	64 ± 9	51/34	I-II	NA
Demelo et al. (25)	RCT	Brazil	62.21 ± 11.34	17/8	1-III	NA
Arfa-Fatollahkhani et al. (13)	RCT	Iran	60/15 ± 8.79	15/5	1-III	NA
Gaßner et al. (20)	RCT	Germany	61.12 ± 6.62	74/26	1-III	NA

USA, United States of America; NA, Not Applicable; M, Male; F, Female.

**Table 2:** Intervention details of the included studies.

Study	Grouping & number	Treatment method	Outcomes
Miyai et al. (18)	BWSTT(10) vs. C3(10)	4 weeks, 3 times/week, BWSTT: 45 min/session, C3: 45 min/session	②⑤
Miyai et al. (17)	BWSTT(11) vs. C3(9)	4 weeks, 3 times/week, BWSTT: 45 min/session, C3: 45 min/session	②⑤
Cakit et al. (39)	TT(21) vs. C1(10)	8 weeks, TT group: 30–35 min/session, C1 group: daily activities	③
Fisher et al. (24)	BWSTT(10) vs. C3(10)	8 weeks, BWSTT: 45 min/session, C3 group: 45 min/session	⑤
Canning et al. (40)	TT(10) vs. C1(10)	6 weeks, 4 times/week, TT group: 30–40 min/session, C1 group: daily activities	①②⑤⑥
Picelli et al. (16)	TT(20) vs. C2(20)	4 weeks, 3 times/week, TT group: 45 min/session, C2 group: 30 min/session	①②③⑤
Harro et al. (19)	TT(10) vs. C2(10)	6 weeks, 3 times/week, TT group: 30 min/session, C2 group: 30 min/session	①③
Ganesan et al. (22)	BWSTT(20) vs. C2(20)	4 weeks, 4 times/week, PWSTT group: 30 min/session, C2 group: 30 min/session	⑤
Ganesan et al. (23)	BWSTT(20) vs. C1(20) vs. C2(20)	4 weeks, 4 times/week, PWSTT group: 30 min/session, C1 group: daily activities, C2 group: 30 min/session	③⑤
Ganesan et al. (21)	BWSTT(20) vs. C1(20) vs. C2(20)	4 weeks, 4 times/week, PWSTT group: 30 min/session, C1 group: daily activities, C2 group: 30 min/session	⑤
Picelli et al. (15)	BWSTT(9) vs. C1(8)	4 weeks, 3 times/week, BWSTT group: 45 min/session, C1 group: daily activities	①②⑤
Cheng et al. (26)	TT(12) vs. C3(12)	4–6 weeks, 3 times/week, TT group: 40 min/session, C3 group: 40 min/session	④⑤⑥
Schenkman et al. (14)	TT(45) vs. C1(40)	26 weeks, 4 times/week, TT: group 40–50 min/session, C1:daily activities	⑤
Demelo et al. (25)	TT(12) vs. C2(13)	4 weeks, 3 times/week, TT: group 20 min/session, C2:20 min/session	①
Arfa-Fatollahkhani et al. (13)	TT(11) vs. C1(9)	10 weeks, 2 times/week, TT group: 30 min/session, C1:daily activities	①④
Gaßner et al. (20)	TT(49) vs. C3(51)	14 days, TTgroup: 25 min/session for 10 individual sessions, C3: 25 min/session for 10 individual sessions	③⑤

NA, Not Applicable; TT, Treadmill Training; BWSTT, Body Weight Supporting Treadmill Training; C1, Daily Training; C2, Gait Training; C3, Comprehensive Training; ①:6MWT,6- min walking test; ②:10MWT,10 Meter Walk Test; ③:BBS, the Berg balance scale; ④: TUG,the time up & go test;⑤: UPDRS III, the unified Parkinson’s disease rating scale part III;⑥:PDQ-39, Parkinson’s Disease Questionnaire-39.

### 3.3 The risk-of-bias assessment

**Figure 2:**
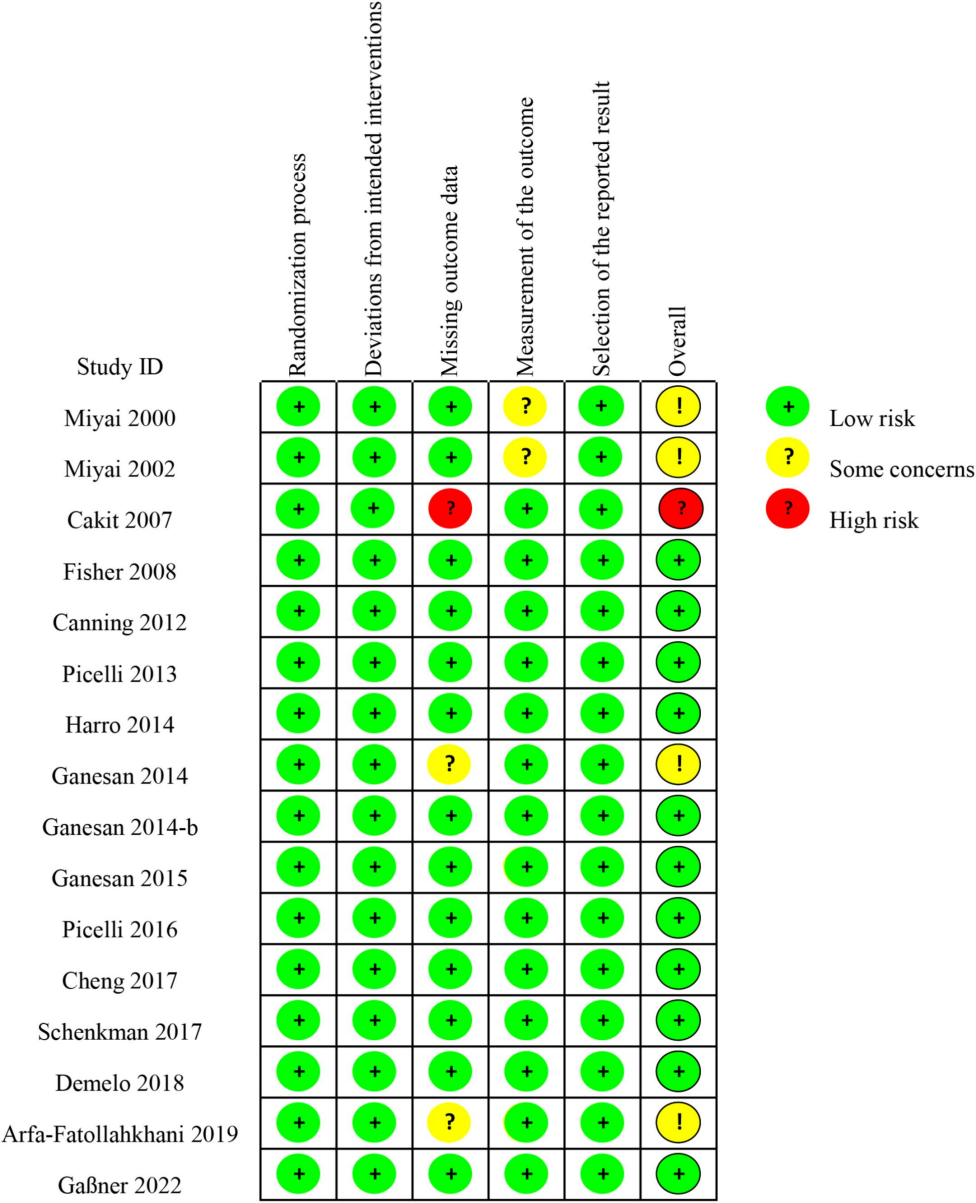
Bias risk assessment summary.

The risk of bias assessment for the included studies indicated that out of the 16 RCT studies11 were considered to be at low risk of bias, 4 had somed some concerns and 1 had a high risk of bias (Figure 2, Supplementary Figure 2).

### 3.4 Outcomes

#### 3.4.1 UPDRS III score

The UPDRS III score is a standardized tool used to evaluate motor function in patients with Parkinson’s disease, where lower scores (SMD) indicate less severe symptoms. Therefore, an SMD (95% CI) < 0 suggests an improvement in symptoms.

In the random effects model, treadmill training significantly reduced the degree of Parkinson’s symptoms compared to conventional training (SMD: -0.45; 95% CI: −0.73 to −0.17), but with greater heterogeneity (I^2^ = 80.2%, *p* = 0.003).

**Figure 3:**
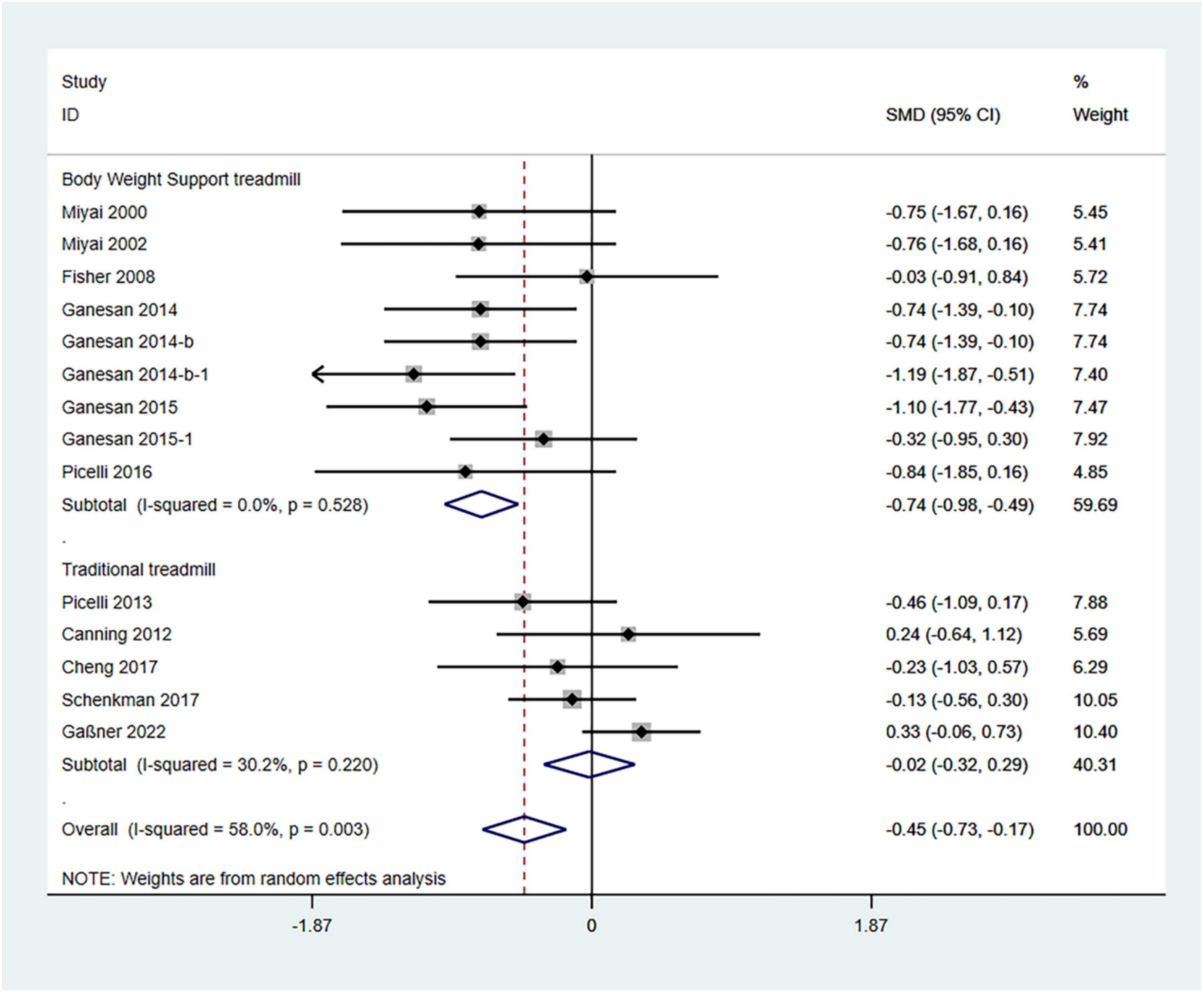
Forest plot of UPDRS III score.

The analysis revealed that the type of treadmill intervention resulted in a high degree of heterogeneity. Subgroup analyses showed that in a random-effects model, BWSTT significantly reduced Parkinson’s symptoms compared with conventional training (SMD: -0.74; 95% CI: −0.98 to −0.49), with low heterogeneity (I^2^ = 0.0%, *p* = 0.528); treadmill training did not show a significant difference in the degree of Parkinsonian symptoms when compared to conventional training (SMD: -0.02; 95% CI: −0.32 to 0.29), and the heterogeneity was moderate (I^2^ = 30.0%, *p* = 0.220) (Figure 3).

The funnel plot and Egger’s test (*p* > 0.05) indicate no publication bias (Supplementary Figures 1–3).

#### 3.4.2 6-MWT

The 6-MWT is primarily used to measure the maximum distance a patient can walk in 6 min and is a widely utilized tool for assessing functional mobility. Higher scores indicate better exercise tolerance. Therefore, an SMD (95% CI) > 0 suggests an improvement in symptoms.

**Figure 4:**
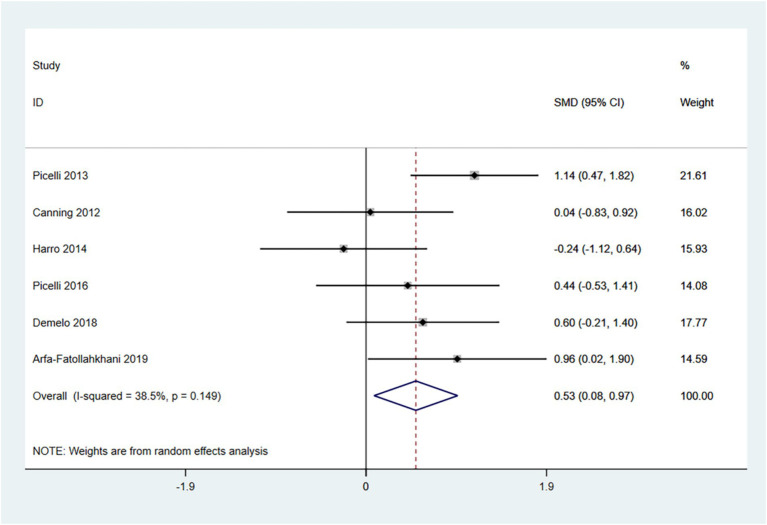
Forest plot of 6-MWT.

In the random effects model, treadmill training significantly increased walking distance compared to conventional training (SMD: 0.53; 95% CI: 0.08 to 0.97), with moderate heterogeneity (I^2^ = 38.5%, *p* = 0.149) (Figure 4).

The funnel plot and Egger’s test (*p* > 0.05) indicate no publication bias (Supplementary Figures 4–6).

#### 3.4.3 10-MWT

The 10-MWT is primarily used to assess short-distance walking speed, balance, and functional mobility. Higher scores indicate better walking ability. Therefore, an SMD (95% CI) > 0 suggests an improvement in symptoms.

In the random effects model, treadmill training significantly increased walking speed compared to conventional training (SMD: 0.93; 95% CI: 0.54 to 1.32), with low heterogeneity (I^2^ = 0.0%, *p* = 0.421).

**Figure 5:**
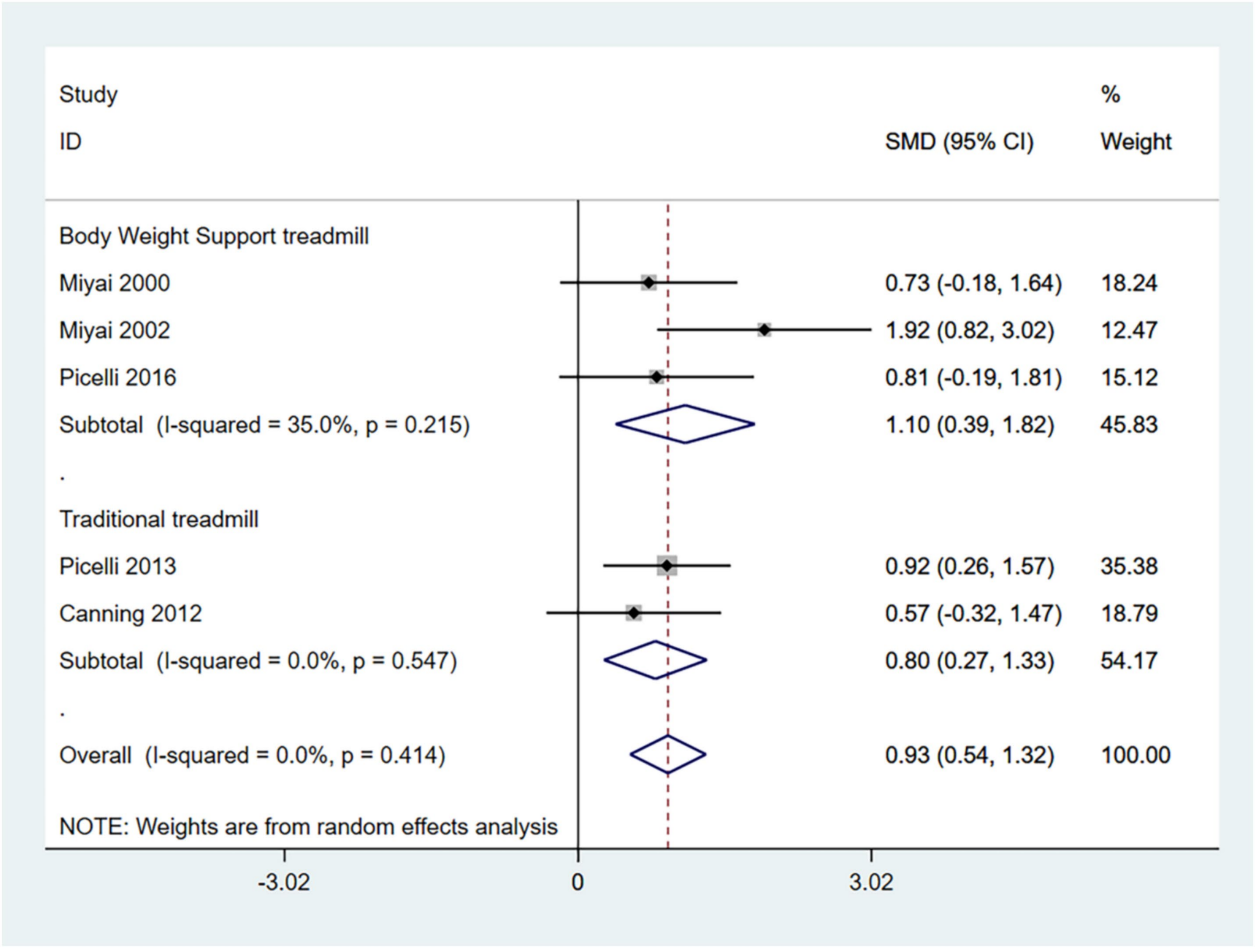
Forest plot of 10-MWT.

In addition, the grouping of interventions revealed that both conventional treadmills and weight-loss treadmills significantly increased walking speed in the 10MWT compared to conventional training. In the random effects model, BWSTT significantly increased walking speed compared to conventional training (SMD: 1.10; 95% CI: 0.39 to 1.82), with moderate heterogeneity (I^2^ = 35.0%, *p* = 0.215); traditional treadmill training significantly increased walking speed compared to conventional training (SMD: 0.80 95% CI: 0.27 to 1.33), with low heterogeneity (I^2^ = 0.0%, *p* = 0.547) (Figure 5). BWSTT is somewhat more effective.

The funnel plot and Egger’s test (*p* > 0.05) indicate no publication bias (Supplementary Figures 7–9).

#### 3.4.4 BBS

The BBS is a comprehensive balance assessment tool that evaluates a patient’s static, dynamic, and functional balance. Higher scores indicate better balance. Therefore, an SMD (95% CI) > 0 suggests an improvement in symptoms.

In the random effects model, treadmill training did not show a significant difference in BBS compared to conventional training (SMD: 0.00; 95% CI: −0.35 to 1.36), with moderate heterogeneity (I^2^ = 48.4%, *p* = 0.085).

**Figure 6:**
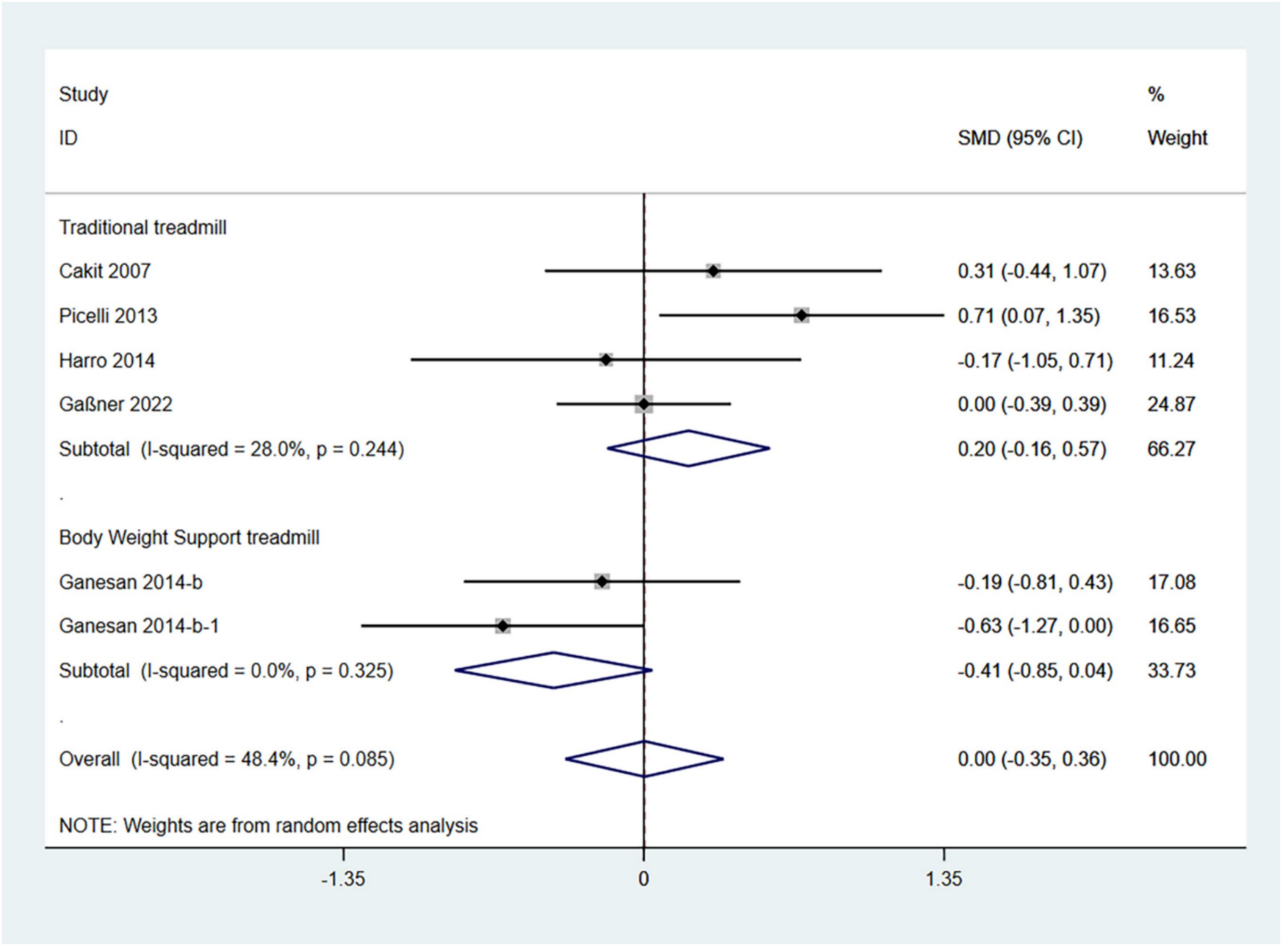
Forest plot of BBS.

The analysis revealed that the type of treadmill intervention resulted in a high degree of heterogeneity. Subgroup analyses showed that in a random-effects model, traditional treadmill training did not show a significant difference in BBS compared to conventional training (SMD: 0.20; 95% CI: −0.16 to 0.57), with moderate heterogeneity (I^2^ = 28.0%, *p* = 0.244); BWSTT did not show a significant difference in BBS compared to conventional training (SMD: -0.41; 95% CI: −0.85 to −0.04), with low heterogeneity (I^2^ = 0.0%, *p* = 0.325) (Figure 6). Traditional treadmill training is somewhat more effective.

The funnel plot and Egger’s test (*p* > 0.05) indicate no publication bias (Supplementary Figures 10–12).

#### 3.4.5 TUG

The TUG comprehensively evaluates an individual’s mobility, balance, and risk of falling. Lower scores (i.e., shorter times to complete the test) indicate that the patient is more mobile, has better balance, and is at a reduced risk of falls. Therefore, an SMD (95% CI) < 0 suggests an improvement in symptoms.

In the random effects model, treadmill training did not show a significant difference in TUG times compared to conventional training (SMD: -0.35; 95% CI: −0.95 to 0.25), with low heterogeneity (I^2^ = 0.0%, *p* = 0.678). Treadmill training has a tendency to reduce TUG times.

**Figure 7:**
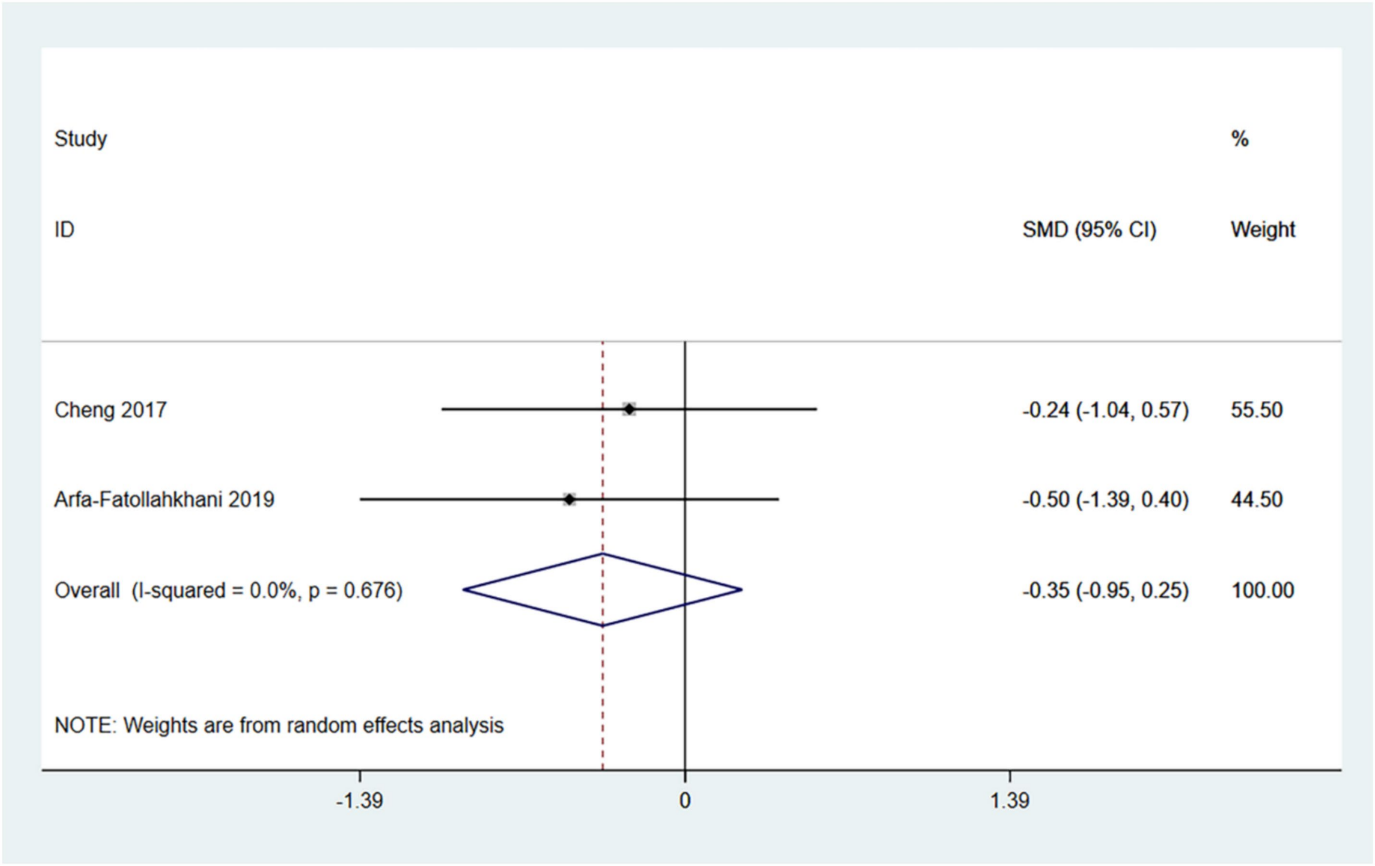
Forest plot of TUG.

The number of articles is insufficient for conducting a funnel plot and Egger’s test (see Figure 7).

#### 3.4.6 PDQ-39

The PDQ-39 provides a comprehensive assessment of the impact of Parkinson’s disease on patients’ daily lives, emotional well-being, and social support. A lower score indicates a better quality of life for the patient. Therefore, an SMD (95% CI) < 0 suggests an improvement in symptoms.

In the random effects model, treadmill training did not show a significant difference in PDQ-39 score compared to conventional training (SMD: -0.35; 95% CI: −0.95 to 0.25), with low heterogeneity (I^2^ = 0.0%, *p* = 0.678). Treadmill training has a tendency to reduce PDQ-39 score.

**Figure 8:**
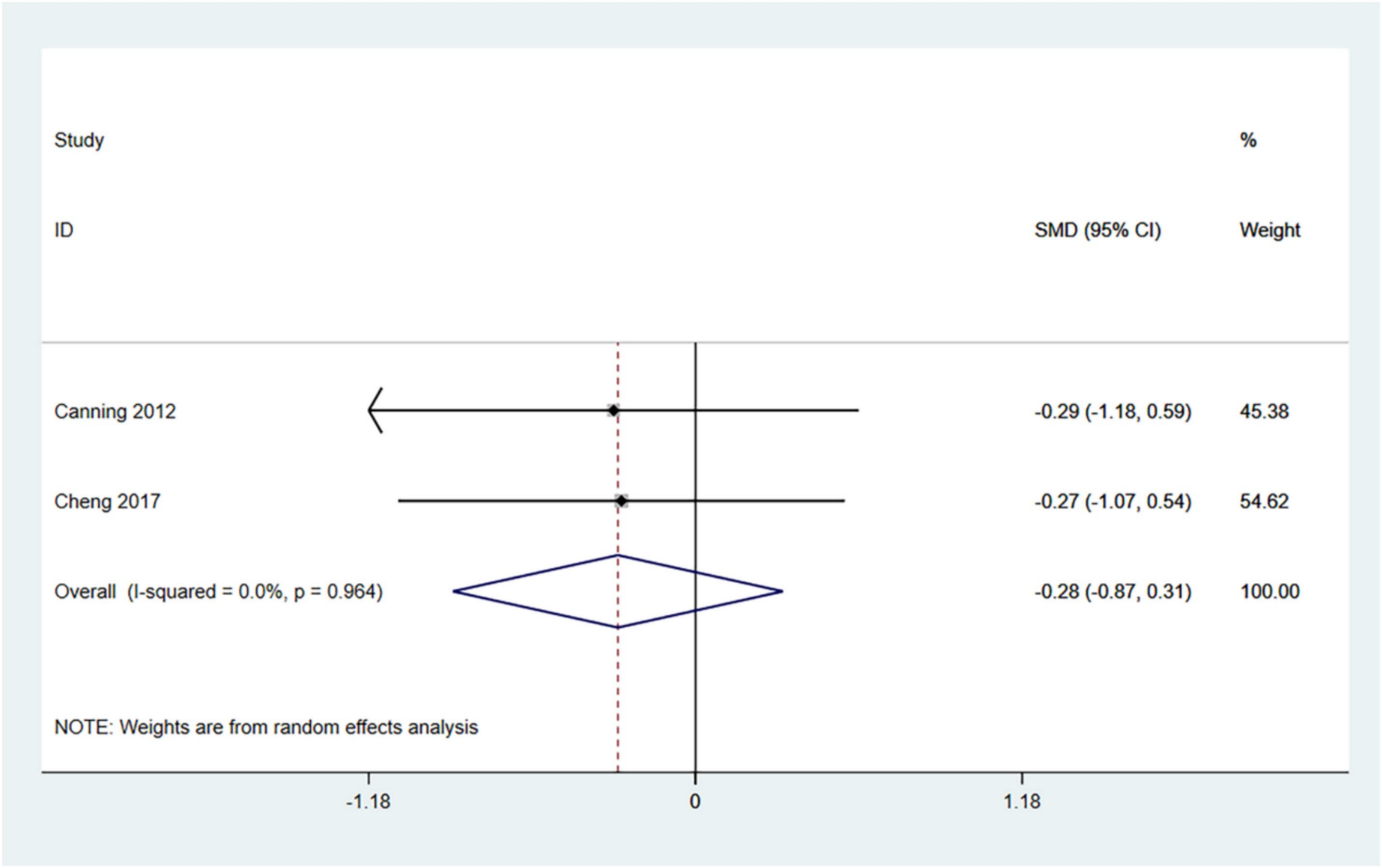
Forest plot of PDQ-39.

The number of articles is insufficient for conducting a funnel plot and Egger’s test (see Figure 8).

### 3.5 Level of evidence

#### 3.5.1 GRADE system recommendation evaluation

Based on the quality assessment and meta-analysis, the GRADE system was employed to evaluate the findings. The results indicated that approximately 66.7% of the evidence was of high quality, while the remaining 33.3% was of moderate quality (Supplementary Table 3).

#### 3.5.2 Oxford Centre for Evidence-Based Medicine: evaluation of the level of evidence

The prevalence of the problem was at level 2, whereas the evaluation of diagnosis, prognosis, treatment benefits, treatment harms, and screening were all assessed at level 1 (Supplementary Table 4).

## 4 Discussion

### 4.1 Discussion of the Main findings

Parkinson’s disease (PD) is a prevalent neurodegenerative disorder characterized by motor dysfunction, impaired balance, and gait abnormalities, which significantly impact patients’ quality of life and independence (27). In recent years, treadmill training has emerged as an effective rehabilitation intervention, demonstrating potential benefits in enhancing motor function and functional capacity in individuals with Parkinson’s disease (28). However, there are still several methodological limitations (e.g., small sample sizes, inconsistent intervention protocols, etc.) and pressing research priorities (e.g., long-term outcome assessment and the optimization of individualized training protocols) in the field. Therefore, this study enhanced its quality through meta-analysis. The results of this meta-analysis indicate that treadmill training, particularly BWSTT, may positively impact lower extremity muscular endurance, cardiorespiratory fitness, functional mobility, and balance coordination in patients with Parkinson’s disease. This improvement may contribute to a reduced risk of falls and enhanced patient independence.

We first analyzed the impact of treadmill training on the enhancement of overall motor abilities in patients with Parkinson’s disease, using the UPDRS III score as a primary index to assess motor function. The results indicated that treadmill training significantly reduced the UPDRS III score compared to conventional training; however, there was a high degree of heterogeneity (I^2^ > 50%). To further investigate the source of this heterogeneity, we conducted a subgroup analysis, categorizing treadmill training into conventional and BWSTT groups. The findings revealed that the BWSTT group significantly reduced the UPDRS III score, accompanied by a notable decrease in heterogeneity. In contrast, the conventional treadmill group did not demonstrate a significant difference in the improvement of the UPDRS III score when compared to conventional therapy. These results suggest that BWSTT is more effective than conventional therapy in enhancing the overall motor function of patients with Parkinson’s disease, while the effects of conventional treadmill training are comparable to those of conventional therapy. By reviewing and analyzing the literature, it appears that the weight-loss treadmill is more effective at reducing the load on the lower limbs during walking (29). This reduction may facilitate walking training for patients, decrease their fear of falling, and boost their motivation and confidence in participating in such training (30). Additionally, it may enhance sensory feedback and promote neuroplasticity by stimulating the motor cortex (31), and such effects could be further augmented by integrating advanced technologies like robotic-assisted gait training and virtual reality tools (32).

This study first assessed the ability of patients with Parkinson’s disease to engage in moderate-intensity exercise over extended periods, as an indicator of their muscular endurance and cardiorespiratory fitness, using the 6-MWT. The results showed that treadmill training significantly increased the walking distance in the 6-MWT compared to conventional rehabilitation methods. Furthermore, the study evaluated short-term walking speed, balance, and functional mobility using the 10-MWT. Treadmill training was associated with a significant improvement in walking speed compared to standard approaches, with the effects of BWSTT appearing even more pronounced.

This study focused on assessing the balance abilities of patients with Parkinson’s disease using BBS. The results indicated that the therapeutic effects of treadmill training and traditional conventional training were comparable in enhancing the balance of these patients. Even after further categorizing treadmill training into conventional and weight-loss groups, the treatment effects in each group remained similar to those observed with conventional routine training. Balance improvements depend not only on lower limb strength but also on sensory systems like proprioception, vision, and vestibular functions (33). As Lena et al. note, rehabilitation interventions target these systems to enhance balance in Parkinson’s disease (34). While treadmill training can effectively enhance gait and lower limb strength, the stimulation of other sensory systems, such as the visual and vestibular systems, may be more limited (35), though recent VR-based interventions have shown promising improvements in these areas (36).

The TUG is utilized to evaluate the functional mobility of patients with Parkinson’s disease. It primarily measures the time required for patients to perform a series of movements, including standing up, walking, turning around, and sitting down, all within a brief period. Analyses revealed no significant difference between treadmill training and traditional conventional training in reducing TUG times. Possible explanations for this finding may include the following: treadmill training primarily focuses on enhancing gait and walking ability, which may have a limited impact on dynamic balance (e.g., turning and one-legged standing) (37). Additionally, the existing literature on this topic is sparse, and the small sample sizes may contribute to a deviation of the results from reality.

Finally, this study utilized the PDQ-39 to assess the quality of life in patients with Parkinson’s disease. The results indicated that treadmill training was comparable to traditional conventional training in enhancing patients’ quality of life. It is hypothesized that this outcome may be attributed to the limited research literature related to this indicator and the insufficient sample size, which may have led to some deviation from the actual results.

### 4.2 Advantages and limitations

This systematic review and meta-analysis offers several advantages:

(1) The substantial number of included studies, the extensive sample size, and the diverse range of countries involved more accurately highlight the advantages of treadmill training over traditional conventional training in enhancing motor function in patients with Parkinson’s disease; (2) Revealed that BWSTT may have superior therapeutic effects; (3) No publication bias was identified in the included studies; (4) revealed potential shortcomings of treadmill training in stimulating the visual and vestibular balance systems; (5) highlighted the potential limitations of treadmill training in enhancing dynamic balance (e.g., turning and one-legged standing; (6) demonstrated the potential benefits of treadmill training in enhancing muscular endurance and short-term walking speed.

This study has several limitations: (1) The study population primarily consists of elderly individuals, which may limit the applicability of the findings to other demographic groups; (2) Regarding the two indicators, TUG test and PDQ-39, the literature included and the sample sizes are relatively small, potentially introducing bias into the results. Further research is necessary to enhance and validate these findings; (3) There are potential shortcomings in the discussion of treadmill training, which are based on a review of the literature. Future experiments should be designed to verify these results; (4) Treadmill training currently lacks a standardized treatment protocol.

### 4.3 Directions for future research

Future research should focus on the following areas: First, efforts should be directed toward further enhancing the treadmill to better meet patients’ needs for visual stimulation and dynamic balance functions, such as turning and one-legged standing. While some studies have started to explore this area, the current research remains insufficient in both breadth and depth. Second, to address the limitations of the small sample size in the current study, future research should include larger cohorts or consider multicenter collaborations to enhance statistical power. it is essential to design large-scale randomized controlled trials to establish standardized protocols. Additionally, research that addresses the humanistic aspects of patient care, such as quality of life, should be incorporated.

Moreover, when considering the practical implementation of treadmill training programs in community or rural settings, it is important to acknowledge several potential barriers. These may include limited access to specialized equipment, a lack of trained professionals, and financial constraints. In rural areas, transportation issues could also pose significant challenges, making it difficult for patients to attend regular sessions. To overcome these obstacles, strategies such as community-based initiatives, telemedicine solutions, and the development of more cost-effective training equipment could enhance the feasibility and effectiveness of treadmill training for Parkinson’s disease patients in these settings, especially when combined with customizable features such as self-selected speeds and immersive VR environments, which have shown clinical benefits (38).

## 5 Conclusion

Compared to traditional training methods, treadmill training offers greater benefits in enhancing lower limb mobility for patients with Parkinson’s disease. It is effective in improving muscular endurance and accelerating short-term walking speed, with weight-loss treadmills demonstrating superior therapeutic efficacy. However, to further enhance the therapeutic effects, improvements in visual stimulation and dynamic balance training on the treadmill are necessary. Additionally, the long-term sustainability and adherence to treadmill training programs should be considered for lasting benefits.

## Data Availability

The original contributions presented in the study are included in the article/Supplementary material, further inquiries can be directed to the corresponding author.
