# Supplementary material for: Treadmill training for gait rehabilitation in elderly patients with mild-to-moderate Parkinson’s disease: a systematic review and meta-analysis
Source: Front Neurol. 2025 Jun 18;16:1609912. doi: 10.3389/fneur.2025.1609912 (PMC12213742; doi:10.3389/fneur.2025.1609912)
Supplement: Supplementary file 1 [file Table_1.docx]

Supplement Table 1 Inclusion and exclusion criteria for the articles

|  | Inclusion Criteria | Exclusion Criteria |
| --- | --- | --- |
| Research object | ①Parkinson's disease Hoehn Yahr clinical stagingⅠ—III |  |
|  | ②mean age±≥55 |  |
|  | ③Disease course≥1year |  |
|  | ④MMSE≥24 |  |
|  | ⑤Stable medical condition |  |
| Intervention measure | ①Traditional or body weight-supported treadmill training | ①Combined with additional interventions |
|  |  | ②Intervention too brief (single/few sessions) |
| Control measure | ①Daily blank, gait, or routine comprehensive training | ①Uneven baseline data |
| Outcome Factor | ①the unified Parkinson's disease rating scale part Ⅲ，UPDRS Ⅲ |  |
|  | ②the Berg balance scale，BBS |  |
|  | ③ the time up & go test，TUG |  |
|  | ④Walking ability：6- minutes walking test，6MWT |  |
|  | ⑤Walking ability：10 Meter Walk Test，10MWT |  |
|  | ⑤Quality of life：the 39/8-item Parkinson's disease questionnaire，PDQ-39 |  |
| Study Design | ①RCT |  |
