# Supplementary material for: Treadmill training for gait rehabilitation in elderly patients with mild-to-moderate Parkinson’s disease: a systematic review and meta-analysis
Source: Front Neurol. 2025 Jun 18;16:1609912. doi: 10.3389/fneur.2025.1609912 (PMC12213742; doi:10.3389/fneur.2025.1609912)
Supplement: Supplementary file 2 [file Table_2.docx]

Supplement Table 2 Risk of bias assessment of the included studies

| Study ID | Random sequence generation | Allocation  concealment | Blinding of participants | Blinding of operator | Blinding of outcome assessment | Incomplete outcome data (report) | Selective reporting  (report) | Other bias |
| --- | --- | --- | --- | --- | --- | --- | --- | --- |
| Miyai 2000 | PY | PY | PN | N | PN | N | N | N |
| Miyai 2002 | PY | PY | PN | N | PN | PN | N | N |
| Cakit 2007 | PY | PY | N | PN | Y | Y | N | N |
| Fisher 2008 | PY | Y | Y | PY | Y | N | N | N |
| Canning 2012 | PY | PY | PY | PN | Y | PN | N | N |
| Picelli 2013 | PN | PY | N | PN | Y | N | N | N |
| Harro 2014 | PN | PY | N | PN | Y | PN | N | N |
| Ganesan 2014 | PY | PY | N | PY | PN | PY | N | N |
| Ganesan 2014-b | PY | PY | N | PY | PN | N | N | N |
| Ganesan 2015 | PY | PY | N | PY | PN | N | N | N |
| Picelli 2016 | Y | Y | N | PY | Y | N | N | N |
| Cheng 2017 | Y | Y | N | PY | Y | N | N | N |
| Schenkman 2017 | Y | Y | N | PY | Y | PN | N | N |
| Demelo 2018 | Y | Y | N | PY | Y | PN | N | N |
| Arfa-Fatollahkhani 2019 | Y | PY | Y | PY | Y | PY | N | N |
| Gaßner 2022 | Y | PY | N | PN | Y | N | N | N |

Note: ID, identification; Y, yes; PY, probably yes; N, no; PN, probably no
