# Supplementary material for: Treadmill training for gait rehabilitation in elderly patients with mild-to-moderate Parkinson’s disease: a systematic review and meta-analysis
Source: Front Neurol. 2025 Jun 18;16:1609912. doi: 10.3389/fneur.2025.1609912 (PMC12213742; doi:10.3389/fneur.2025.1609912)
Supplement: Supplementary file 3 [file Table_3.docx]

**Supplement Table3 GRADE system recommended rating results**

|  | Quality evaluation | | | | | | outcome | | | |
| --- | --- | --- | --- | --- | --- | --- | --- | --- | --- | --- |
| Index | Risk of bias | Discordance | Indirectness | Inaccuracy | Publication bias |  | Articles（patients) | SMD(95%Cl) | quality | recommend |
| UPDRS III | A | A | A | A | A |  | 14 (546) | -0.45 (-0.73, -0.17) | high | strong |
| 6MWT | A | A | A | A | A |  | 6 (142) | 0.53 (-0.08, 0.97) | high | strong |
| 10MWT | A | A | A | A | A |  | 5 (117) | 0.93 (0.54, 1.32) | high | strong |
| BBS | A | A | A | A | A |  | 6 (271) | 0.00 (-0.35, 0.36) | high | weak |
| TUG | A | A | A | B ^a^ | A |  | 2 (44) | -0.35 (-0.95, -0.25) | medium | weak |
| PDQ-39 | A | A | A | B ^a^ | A |  | 2 (44) | -0.28 (-0.87, 0.31) | medium | weak |

Note：A, no downgrade; B, downgrade 1; a, Small sample size or wide CI range;UPDRS III, the unified Parkinson's disease rating scale part Ⅲ; BBS, the Berg balance scale; TUG, the time up & go test; 6MWT, 6- minutes walking test; 10MWT, 10 Meter Walk Test; PDQ-39, the 39/8-item Parkinson's disease questionnaire.
