# Supplementary material for: Treadmill training for gait rehabilitation in elderly patients with mild-to-moderate Parkinson’s disease: a systematic review and meta-analysis
Source: Front Neurol. 2025 Jun 18;16:1609912. doi: 10.3389/fneur.2025.1609912 (PMC12213742; doi:10.3389/fneur.2025.1609912)
Supplement: Supplementary file 4 [file Table_4.docx]

**Supplement Table4 Oxford centre for evidence-based medicine 2011 levels of evidence**

| Question | Step 1  (Level 1*) | Step 2  (Level 2*) | Step 3  (Level 3*) | Step 4  (Level 4*) | Step 5 (Level 5) | Evaluation results |
| --- | --- | --- | --- | --- | --- | --- |
| How common is the problem? | Local and current random sample surveys (or censuses) | Systematic review of surveys that allow matching to local circumstances** | Local non-random sample** | Case-series** | n/a | Level 2 |
| Is this diagnostic or monitoring test accurate?  (Diagnosis) | Systematic review  of cross sectional studies with consistently applied reference standard and blinding | Individual cross sectional studies with consistently applied reference standard and blinding | Non-consecutive studies, or studies without consistently applied reference standards** | Case-control studies, or “poor or non-independent reference standard** | Mechanism-based reasoning | Level 1 |
| What will happen if we do not add a therapy?  (Prognosis) | Systematic review  of inception cohort studies | Inception cohort studies | Cohort study or control arm of randomized trial* | Case-series or case- control studies, or poor quality prognostic cohort study** | n/a | Level 1 |
| Does this intervention help?  (Treatment Benefits) | Systematic review  of randomized trials or n-of-1 trials | Randomized trial or observational study with dramatic effect | Non-randomized controlled cohort/follow-up study** | Case-series, case-control studies, or historically controlled studies** | Mechanism-based reasoning | Level 1 |
| What are the COMMON harms?  (Treatment Harms) | Systematic review of randomized trials, systematic review of nested case-control studies, n- of-1 trial with the patient you are raising the question about, or observational study with dramatic effect | Individual randomized trial or (exceptionally) observational study with dramatic effect | Non-randomized controlled cohort/follow-up study (post-marketing surveillance) provided there are sufficient numbers to rule out a common harm. (For long-term harms the duration of follow-up must be sufficient.)** | Case-series, case-control, or historically controlled studies** | Mechanism-based reasoning | Level 1 |
| What are the RARE harms?  (Treatment Harms) | Systematic review of randomized trials or n-of-1 trial | Randomized trial or (exceptionally) observational study with dramatic effect |  |  |  | Level 1 |
| Is this (early  detection) test  worthwhile?  (Screening) | Systematic review of randomized trials | Randomized trial | Non -randomized controlled cohort/follow-up study** | Case-series, case-control, or historically controlled studies** | Mechanism-based reasoning | Level 1 |
