# Supplementary material for: Treadmill training for gait rehabilitation in elderly patients with mild-to-moderate Parkinson’s disease: a systematic review and meta-analysis
Source: Front Neurol. 2025 Jun 18;16:1609912. doi: 10.3389/fneur.2025.1609912 (PMC12213742; doi:10.3389/fneur.2025.1609912)
Supplement: Supplementary file 5 [file Supplementary_file_1.docx]

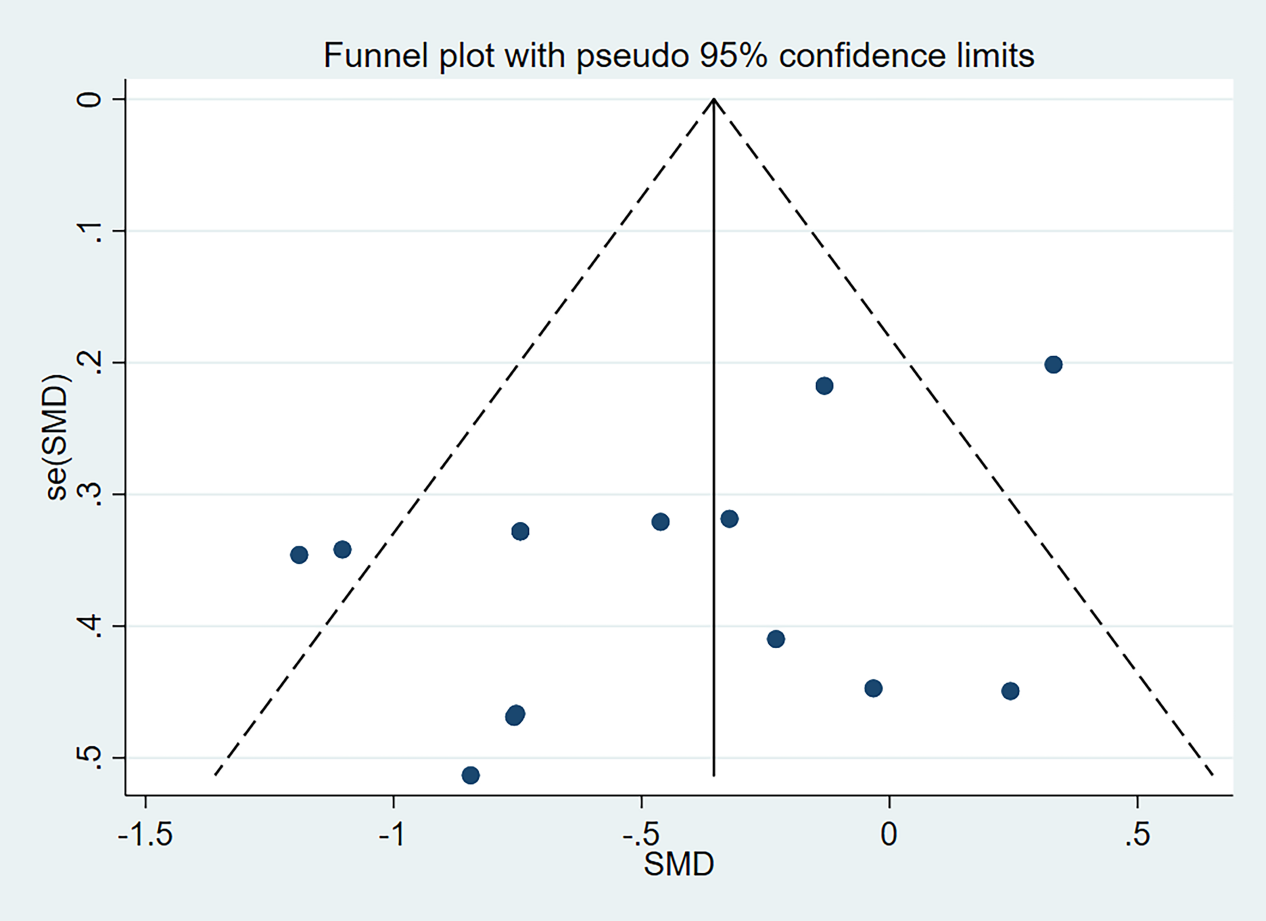


Supplement Figure 1 Funnel plot of UPDRS III score


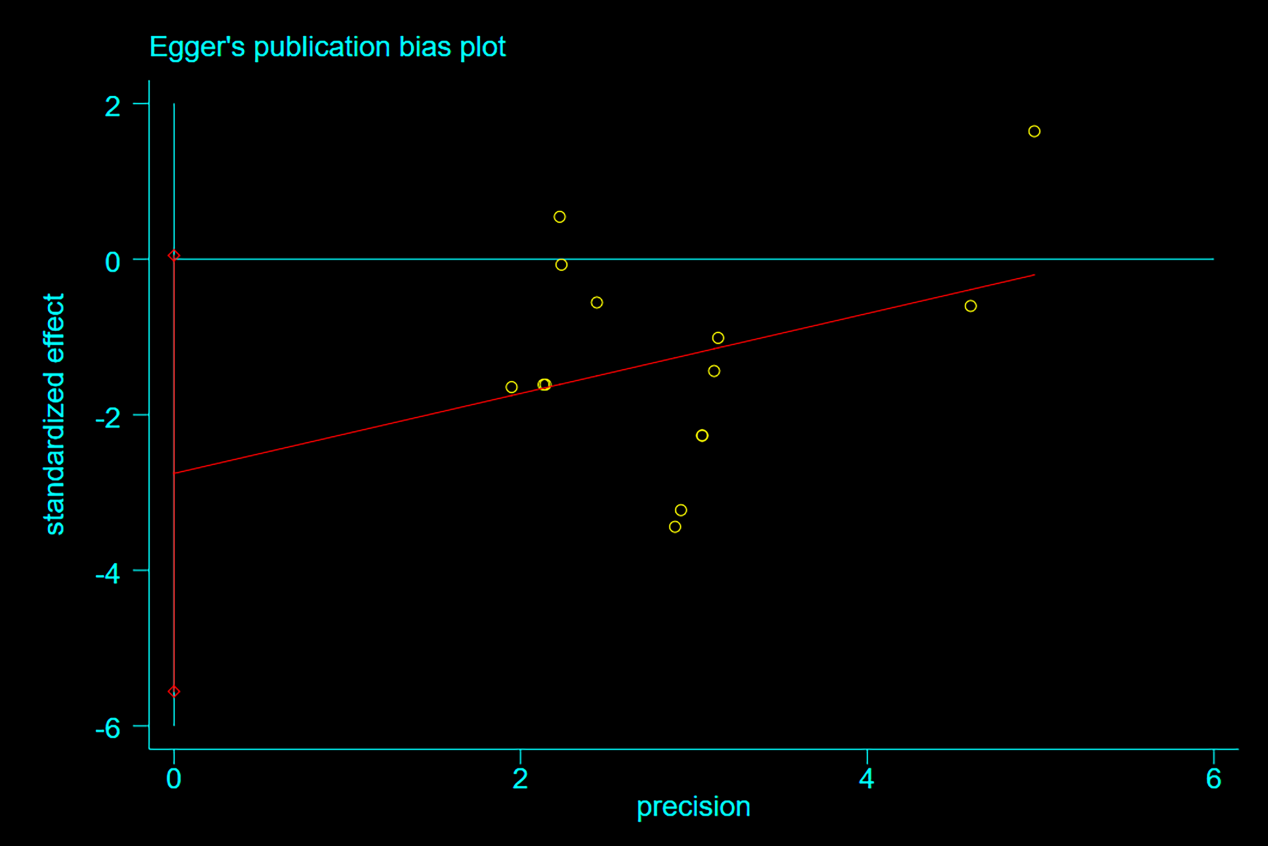
Supplement Figure 2 Egger's test of UPDRS III score


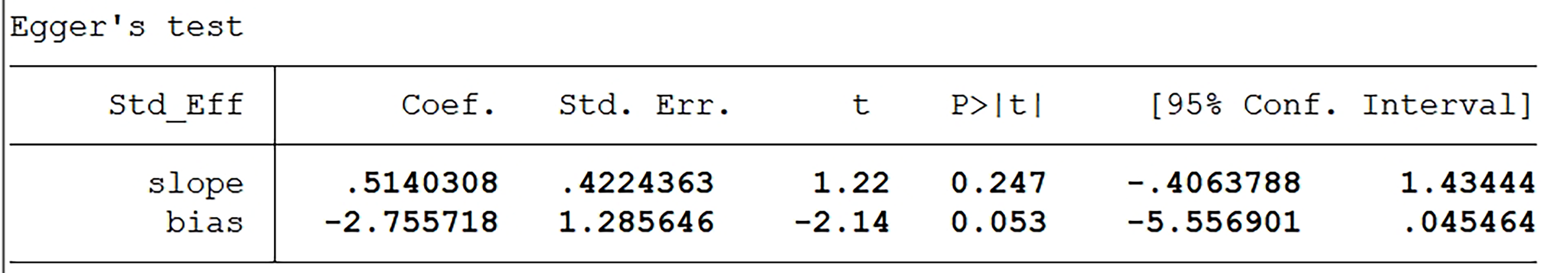


Supplement Figure 3 Egger's test of UPDRS III score (numerical value)

Supplement Figure 4 Funnel plot of 6-MWT

Supplement Figure 5 Egger's test of 6-MWT


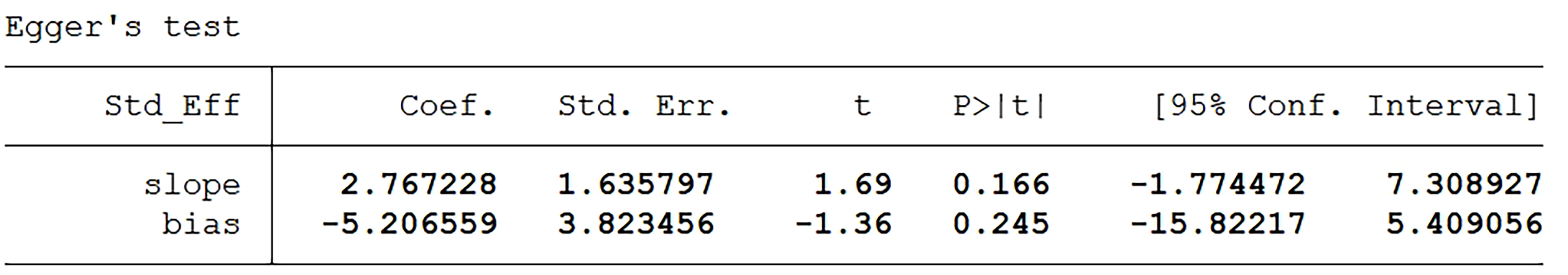


Supplement Figure 6 Egger's test of 6-MWT (numerical value)


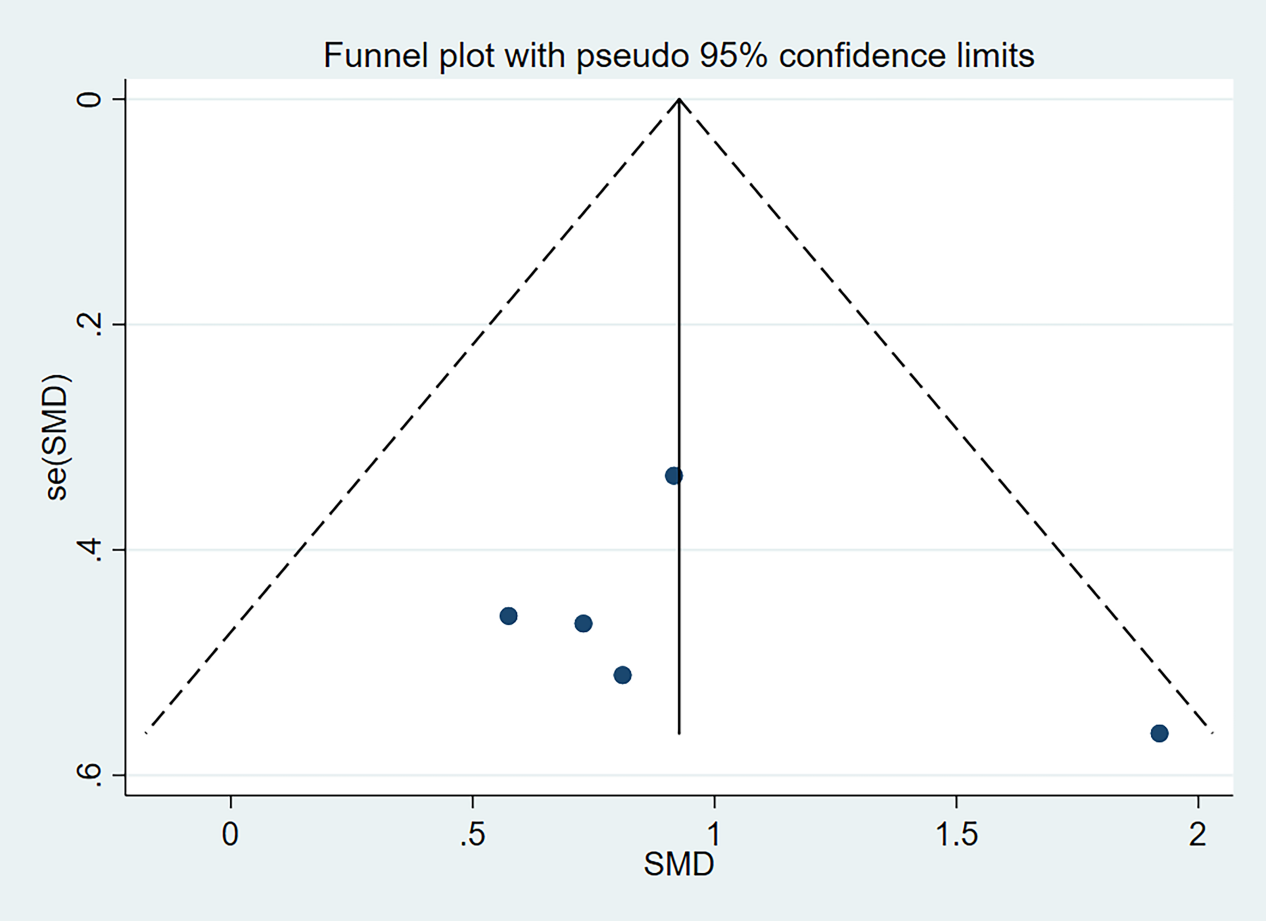
Supplement Figure 7 Funnel plot of 10-MWT


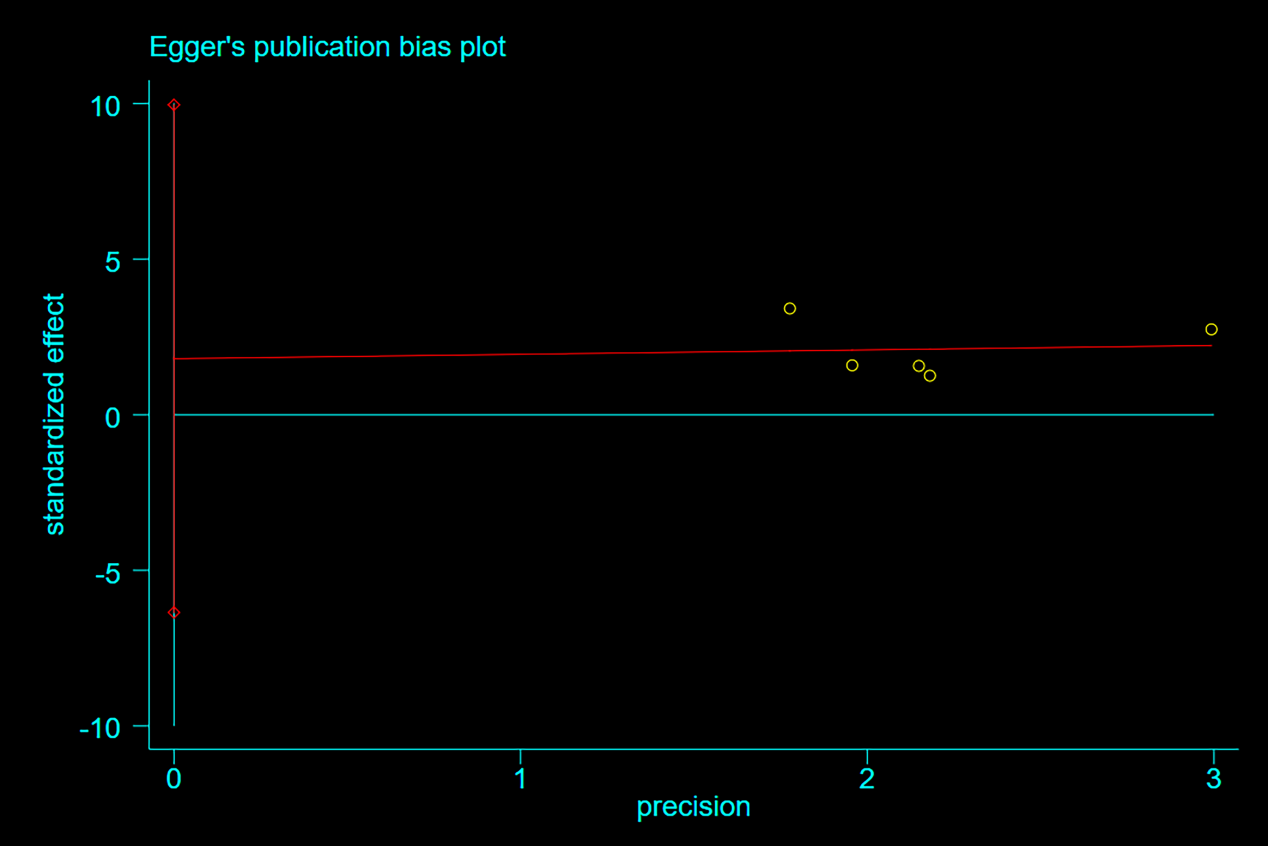
Supplement Figure 8 Egger's test of 6-MWT


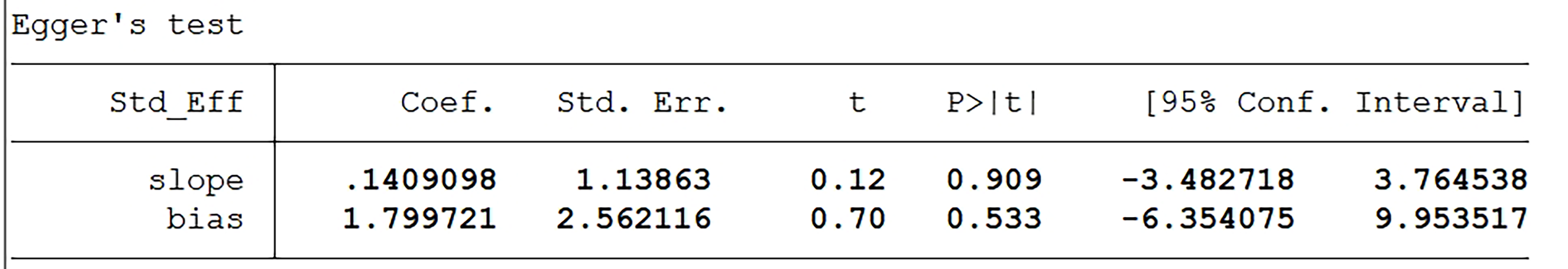


Supplement Figure 9 Egger's test of 10-MWT (numerical value)


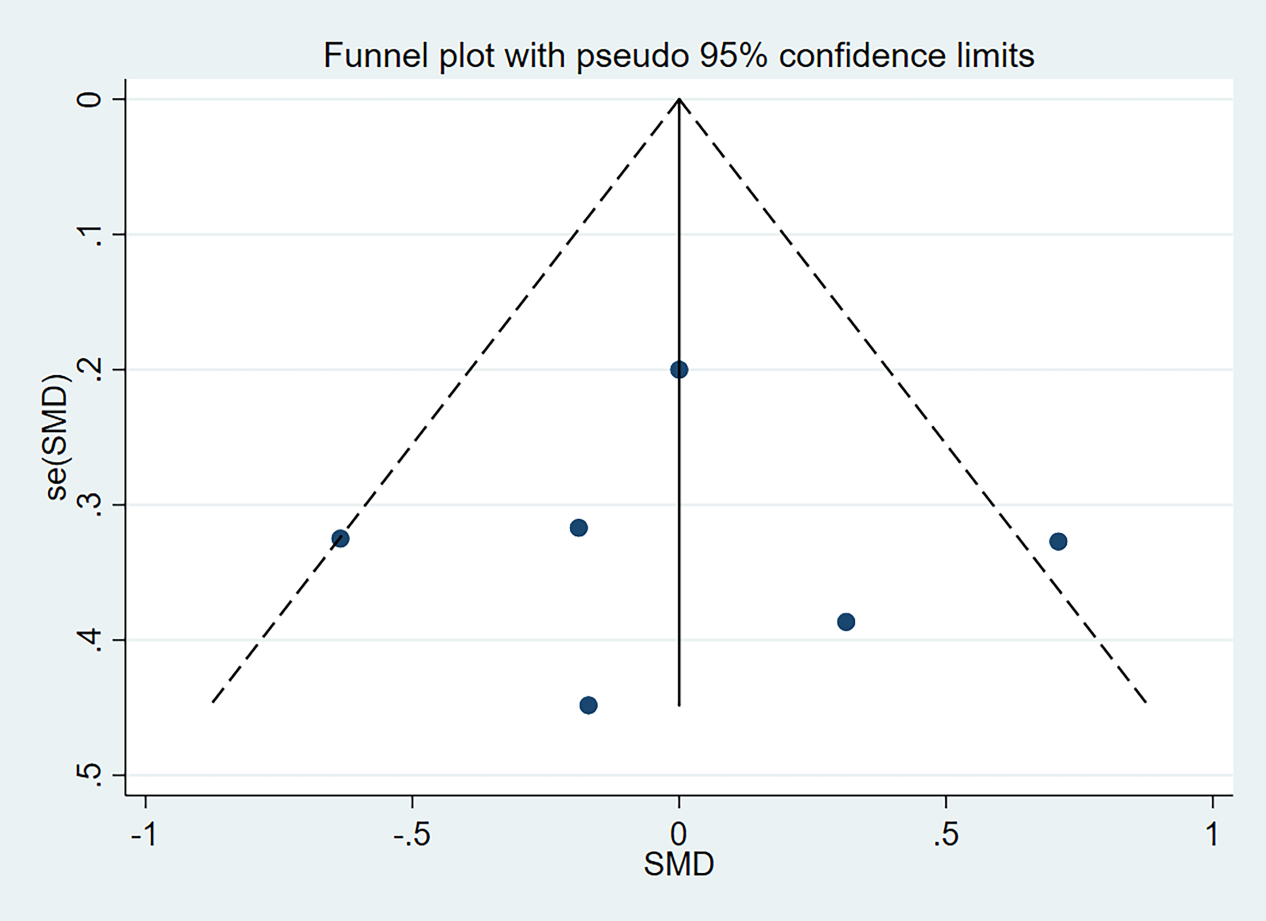
Supplement Figure 10 Funnel plot of BBS


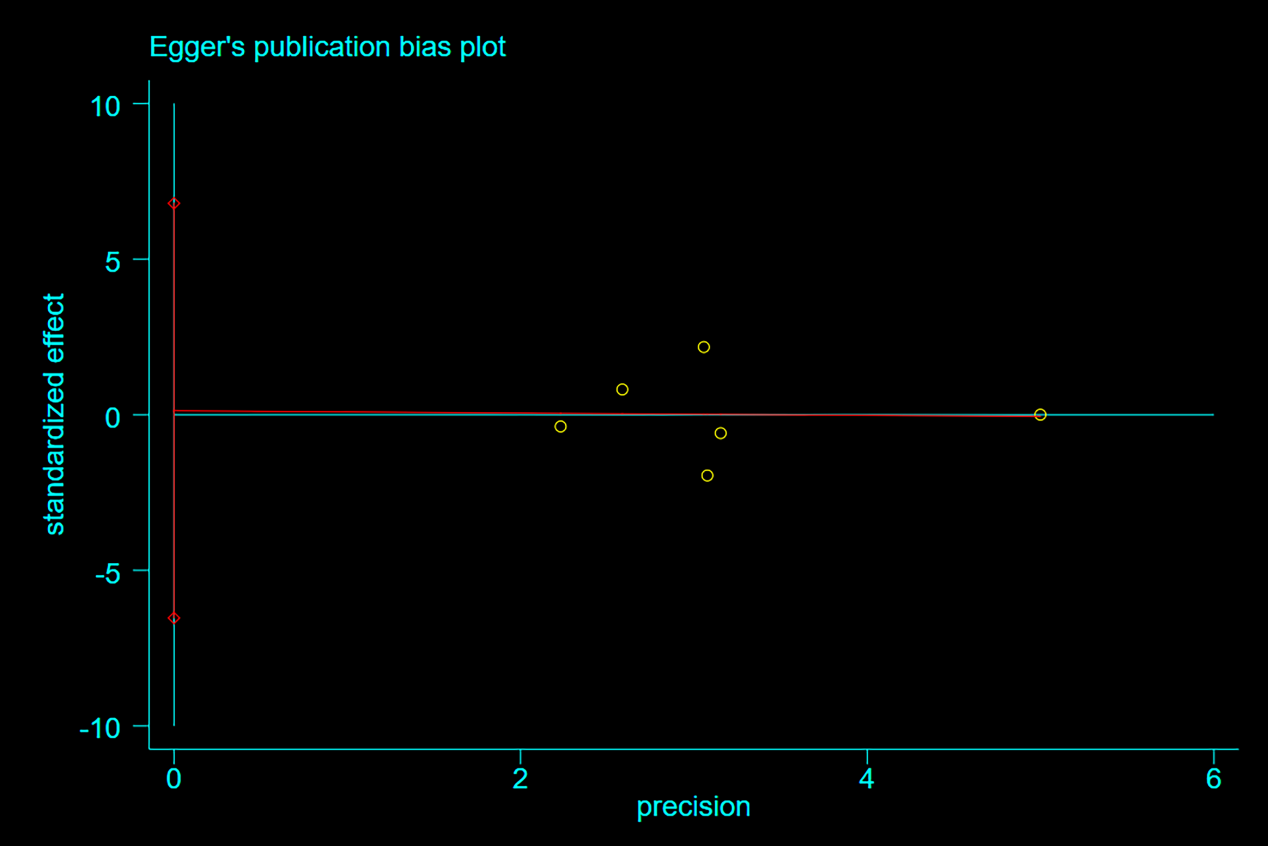


Supplement Figure 11 Egger's test of BBS


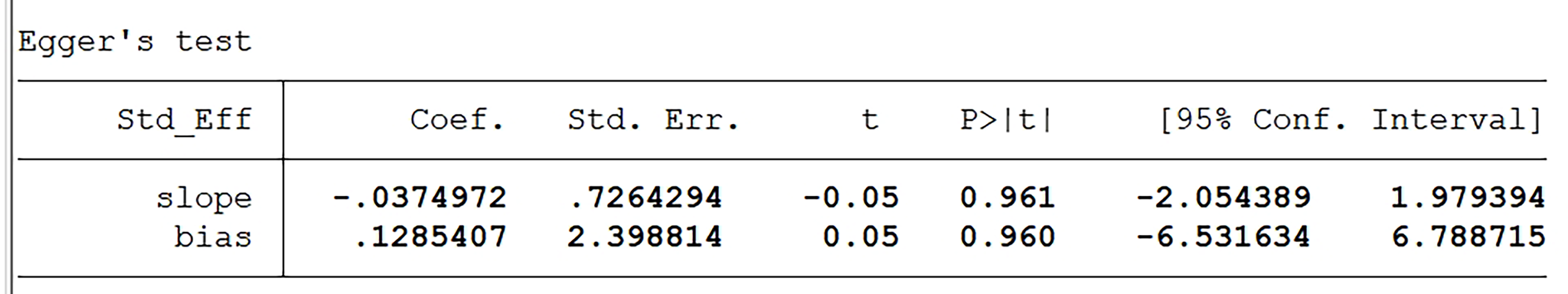


Supplement Figure 12 Egger's test of BBS
